# Association between biomarkers of iron status and cardiometabolic risk in Spanish children aged 9–10 years. The ELOIN study

**DOI:** 10.1007/s00431-023-05244-1

**Published:** 2023-10-11

**Authors:** Honorato Ortiz-Marrón, Gloria Cabañas Pujadas, Encarnación Donoso Navarro, Mar Burreros García, María Isabel Herreros Álvaro, Alma María Mejía Fernández de Velasco, Ana Cornejo Gutiérrez, Iñaki Galán

**Affiliations:** 1 Cardiovascular Disease Surveillance Technical Unit, Directorate-General of Public Health. Ministry of Health of Community of Madrid, Madrid, Spain; 2 Clinical Analysis and Biochemistry Service, Puerta de Hierro University Hospital, Madrid, Majadahonda Spain; 3 Health Center Collado Mediano. Collado Mediano, Ministry of Health of Community of Madrid, Madrid, Spain; 4 Health Center Daroca, Ministry of Health of Community of Madrid, Madrid, Spain; 5 Health Center Aranjuez. Aranjuez, Ministry of Health of Community of Madrid, Madrid, Spain; 6 Health Center Barcelona. Móstoles. Ministry of Health of Community of Madrid, Madrid, Spain; 7 National Centre for Epidemiology, Institute of Health Carlos III. Madrid, Madrid, Spain; 8 Department of Preventive Medicine and Public Health. Faculty of Medicine, Universidad Autónoma de Madrid (IdiPaz), Autonomous University of Madrid), Madrid, Spain

**Keywords:** Iron markers, Ferritin, Transferrin, Transferrin saturation, Cardiac risk, Children

## Abstract

The relationship between iron metabolism and cardiometabolic risk factors has been scarcely studied in children, and the results are controversial. The objective of this study was to evaluate the association between iron parameters and lipid, glycemic and blood pressure alterations in the pediatric population. This was a cross-sectional study of 1954 children between 9 and 10 years of age in Madrid (Spain), participants in a longitudinal study of childhood obesity. Iron metabolism parameters, i.e., serum iron (*Is*), ferritin (*Fs*), transferrin (*Tf*) and transferrin saturation (*STf*) and lipid, glycemic and blood pressure profiles were evaluated. Odds ratios (ORs) were estimated using logistic regression models adjusted for sociodemographic characteristics, diet, physical activity, C-reactive protein and body mass index. Compared with the participants in the low *Is* and *STf* tertiles, those in the upper tertiles had a lower risk of low HDL-Chol (OR: 0.34; 95%CI: 0.17; 0.67) and OR: 0.44 (95%CI: 0.23**;** 0.84), respectively, and children in the upper *Fs* tertile had an OR of 2.07 (95%CI: 1.16**;** 3.68) for low HDL-Chol. Children in the highest *Is* and *STf* tertiles had a lower risk of prediabetes [OR: 0.63 (95%CI: 0.41**;** 0.97) and OR: 0.53 (95%CI: 0.34; 0.82)] and insulin resistance (IR) (OR: 0.37; 95%CI: 0.22**;** 0.64), and those in the upper *Tf* tertile had a higher risk of IR (OR: 1.90; 95%CI: 1.16; 3.12). An increased risk of hypertension was found only in children in the upper *Fs* tertile (OR: 1.46; 95%CI: 1.01; 2.13).

* Conclusions*: Biomarkers of iron metabolism are associated with cardiometabolic alterations in the pediatric population, with a variable direction and magnitude depending on the indicators used.

**Table:** 

**What is Known:** *• Iron metabolism is related to important cardiometabolic alterations such as metabolic syndrome and its components.* *• Association between biomarkers of iron status and cardiometabolic risk have been less explored in children.*	
**What is New:** *• Biomarkers of iron metabolism are associated with cardiometabolic alterations in the pediatric population.* *• Iron parameters in the pediatric population could be of great help to detect and prevent cardiometabolic abnormalities early.*

**Supplementary Information:**

The online version contains supplementary material available at 10.1007/s00431-023-05244-1.

## Introduction

Iron is a necessary mineral for the human body and plays an important role in essential biological processes such as DNA synthesis, haemoglobin formation, and oxygen transport [1]. Iron homeostasis is maintained through complex mechanisms involved in intake, transfer and storage. Ferritin is an iron storage protein; therefore, high concentrations of serum ferritin indicate a risk of iron overload. Furthermore, ferritin is an acute phase protein that can be affected by other infectious and metabolic processes [2]. Transferrin, or transporter protein, increases when iron requirements increase, and its saturation reflects the amount of iron it is capable of fixing. When transferrin is saturated, free iron remains, which is toxic to cells [3].

Inappropriate overloads or deficiencies of this mineral can contribute to metabolic disturbances that correlate with a wide range of cardiovascular diseases and type 2 diabetes [4–7]. Iron deficiency is common in patients with heart failure and pulmonary hypertension [5, 8], and iron overload is related to an increased risk of atherosclerosis as well as the appearance of metabolic syndrome (MetS) and its components, such as dyslipidemia, increased body mass index (BMI), high blood pressure, hyperglycemia and insulin resistance [9–11].

However, most of the scientific evidence comes from studies carried out in the adult population; therefore, it is not clear whether these associations occur in early life and what effects changes in iron levels and cardiometabolic risk have in early life. Studies that explore this relationship in children are limited, and their results are inconclusive [12–14].

Cardiometabolic risk factors (CMRFs) usually appear in an aggregate way, and the coexistence of 3 or more risk factors is defined as MS [15]. For the preschool and childhood stages, there is no accepted definition of MetS because in this period, there are intrinsic variations in age and growth; therefore, it is more appropriate to analyze the components of MetS and other CMRFs independently [16].

The objective of this study was to explore the association between four iron parameters, i.e., serum iron (*Is*), serum ferritin (*Fs*), serum transferrin (*Tf*) and transferrin saturation (*STf*), and CMRFs in the child population aged 9–10 years.

## Methods

### Study population

The data were obtained from the Longitudinal Study of Childhood Obesity (ELOIN study), a population-based prospective longitudinal study consisting of a representative baseline cohort of the 4-year-old population of the Community of Madrid (6.8 million inhabitants); the methodology has been previously published [17]. The study consisted of a physical examination of children at 4, 6, and 9 years of age and a telephone interview with the parents, as well as the collection of a blood sample at 9 years of age.

*Inclusion and exclusion criteria:* For this cross-sectional study, 1954 participants aged 9–10 years old (born in 2008–2009) and who had a physical examination a blood sample and parent interview were included. On the other hand, 1030 children who had physical examination but not blood sample data were excluded and 8 children who had blood sample but not physical examination data were also excluded.

### Blood pressure and anthropometric measurements

Physical examinations were performed by pediatricians and nurses from the Sentinel Network of the 31 primary care centers participating in the study [18]. Standardized measurements of weight, height and blood pressure (BP) were collected.

BP was measured in pediatric offices using the auscultatory method with the right arm. The participants remained seated at rest for 5 min. Two measurements were taken at least 2 min apart on the same day, and a third measurement was repeated if there were differences over 4 mmHg. The average of the measurements was used. BP values were standardized by age, sex and height using the reference tables of the Fourth Report of Diagnosis, Evaluation, and Treatment of High Blood Pressure in Children and Adolescents (NHBPEP) [19].

Weight was measured by means of a digital scale, and height was measured with a telescopic height rod. Two measurements were made, and the average was calculated. Using the weight and height, BMI was estimated in kg/m^2^.

Biochemical parameters were obtained from a blood sample taken at primary care centers by venipuncture using a Vacutainer©; the samples were obtained after the participant had fasted for 8 h. Venous blood was collected and separated into a tube with anticoagulant and a tube with whole blood, and the samples were stored at 4 °C immediately after extraction.

The following serum parameters of iron metabolism were analyzed: serum iron (*Is*) by ferrocin, serum ferritin (*Fs*), serum transferrin (*Tf*) and transferrin saturation (*STf*). *STf* values were calculated using the following formula: *STf* % = (Serum iron (µg/dl) × 70,645)/Transferrin (mg/dl) [20].

Total cholesterol (TC) and high-density lipoproteins (HDL-Chol) were quantified using enzymatic methods (cholesterol oxidase, esterase and peroxidase); low-density lipoproteins (LDL-Chol) were quantified using the Friedewald formula: LDL = (CT-HDL)—(TG/5); triglycerides (TG) were quantified using by the lipase/glycerol kinase colorimetric method; C-reactive protein (CRP) was quantified via immunoturbidimetry; and glucose was quantified using an enzymatic method (glucose hexokinase coupled to glucose 6-P dehydrogenase). All measurements were carried out on an ADVIA Chemistry XPT from Siemens Healthineers in the Clinical Biochemistry Laboratory of Puerta de Hierro University Hospital (Majadahonda, Madrid).

Insulin was measured via chemiluminescence, and HbA1c was measured via high-performance liquid chromatography (HPLC) on a TOSOH G8 analyzer. Insulin resistance (IR) was estimated using the homeostatic model assessment of insulin resistance (HOMA-IR), calculated as (fasting glucose (mg/dL) x fasting insulin (µU/mL))/405 [21].

### Definition of metabolic abnormalities

The criteria of the National Heart, Lung and Blood Institute [22] and the European Guidelines on Cardiovascular Disease Prevention [23] were used to define altered lipid and glycemic profile values:

Lipid profile: high TC, TC ≥ 200 mg/dL; high TG, TG ≥ 130 mg/dL; low HDL-Chol, HDL-Chol < 40 mg/dL; and high LDL-Chol, LDL-Chol ≥ 130 mg/dL; and.

Glycemic profile: prediabetes, fasting blood glucose > 100 mg/dL or HbA1c ≥ 5.7%; hyperinsulinemia ≥ 15 µU/dL; and high IR, HOMA-IR ≥ 3.16 µU/dL.

High BP was defined using the 90th percentile of systolic and/or diastolic BP for age and sex following the recommendations of the European Society of Hypertension for children and adolescents [24].

### Covariates

The sociodemographic variables included sex, age, and family purchasing power. Purchasing power was estimated with the *Family Affluence Scale* (FAS-II), which is a global indicator of a family's socioeconomic level [low (0–3 points), medium (4–5 points) or high (6–9 points)] [25].

Other covariates were mean daily iron intake; quality of the diet (*Mediterranean Diet Quality Index* (Med-DQI) [26] estimated on the basis of a semiquantitative food consumption frequency questionnaire used to record the frequency of consumption (daily, weekly, monthly or yearly) of 145 food items in the last year); and physical activity (*Physical Activity Questionnaire—Children* (PAQ-C), with a score ranging from 1 (little physical activity) to 5 (high physical activity)) [27].

This study complies with the Declaration of Helsinki. The study protocol was approved by the Ethics Committee of Ramón y Cajal University Hospital in Madrid, Spain (CIHURC- 122/11). Written consent was obtained from the parents and/or guardians of the participants, and the data were anonymized to ensure confidentiality.

### Statistical analysis

*Is* (< 68, 69–94 and > 94 μg/mL)*, Fs* (< 33, 33–49 and > 49 ng/mL), *Tf* (< 256, 256–280 and > 280 mg/dl) and *STf* (< 17.8, 17.8–24.5 and > 24.5%) were categorized into tertiles.

To study the association between iron metabolism markers and cardiometabolic biochemical parameters, two sequential multiple linear regression models were developed, with progressive adjustment of the variables, using the lower tertile as the reference category: Model 1) adjusted for sex, age, family purchasing power, iron intake, quality of diet and physical activity; and Model 2) Model 1 plus BMI and CRP. Taking into account that some markers, mainly glycemic markers, did not follow a normal distribution, these linear regression models were repeated with logarithmic transformation of the cardiometabolic biochemical parameters (supplementary material).

Through logistic regression, associations between the tertiles of the markers of iron metabolism and the cardiometabolic alterations and adjusting for the possible confounding factors were estimated, calculating odds ratios (ORs) with the same sequential models described above.

The level of statistical significance was established at p < 0.05 (two-tailed) for all estimators.

The analyses were carried out in STATA 16.1 (StataCorp, College Station, Texas, USA).

## Results

Table 1 provides the demographic and anthropometric characteristics and mean values for biomarkers of iron metabolism and the lipid and glycemic profiles of the study population (N = 1954). The mean age of the participants was 9.0 years, and 51.5% were female. A total of 14.0% lived in households with low purchasing power. The mean concentrations of *Is, Fs, Tf, and STf* were 83.0 μg/mL, 45.5 ng/mL, 269.5 mg/dL, and 22.0%, respectively.

**Table 1:** Description of the study population

	n	Mean (SD) / %
Age (years), mean (SD)	1954	9.0 (0.4)
Sex, %		
Male	947	48.5
Female	1007	51.5
Family purchasing power^a^, %		
Low	264	14.0
Medium	432	22.9
High	1190	63.1
Height (cm), mean (SD)	1954	136.4 (6.6)
Weight (kg), mean (SD)	1954	34.0 (7.9)
Body mass index (kg/m^2^), mean (SD)	1954	18.1 (3.2)
Total cholesterol (mg/dL), mean (SD)	1954	164.1 (26.2)
HDL cholesterol (mg/dL), mean (SD)	1954	60.2 (13.6)
LDL cholesterol (mg/dL), mean (SD)	1954	90.8 (22.5)
Triglycerides (mg/dL), mean (SD)	1954	65.3 (31.7)
Glucose (mg/dL), mean (SD)	1950	83.8 (7.5)
Glycated hemoglobin (%), mean (SD)	1945	5.3 (0.3)
Insulin (μU/mL), mean (SD)	1912	7.8 (7.7)
HOMA-IR^b^ (µU/dL), mean (SD)	1909	1.7 (1.9)
Systolic pressure (mmHg), mean (SD)	1952	97.2 (11.2)
Diastolic pressure (mmHg), mean (SD)	1951	58.4 (9.0)
C-Reactive protein (mg/dl), mean (SD)	1954	1.76 (6.0)
Serum iron (μg/mL), mean (SD)	1954	83.0 (30.3)
Serum ferritin (ng/mL), mean (SD)	1954	45.5 (25.2)
Transferrin (mg/dl), mean (SD)	1954	269.5 (29.9)
Transferrin saturation (%), mean (SD)	1954	22.0 (8.6)
Dietary iron intake (g/day), mean (SD)	1896	13.9 (3.9)
Diet quality score^c^, mean (SD)	1896	6.4 (1.7)
Physical activity^d^, mean (SD)	1895	3.1 (0.69)

*SD *standard deviation

^a^Measured through the Family Affluence Scale (FAS-II)

^b^Homeostatic Model Assessment–Insulin Resistance

^c^Mediterranean–Diet Quality Index (Med–DQI), scores range from 1 to 14

^d^Physical Activity Questionnaire-Children (PAQ-C), scores range from 1-5

Tables 2 and 3 show the results of the multiple linear regression models for *Is*, *Fs*, *Tf* and *STf* with the cardiometabolic parameters, adjusted for the main covariates. In Model 2, controlling for sociodemographic variables, diet quality, iron intake, physical activity, BMI and CRP, participants with high levels of *Is* (tertile 3), compared to those with low levels (tertile 1), had higher CT and HDL-Chol values, with estimated β coefficients of 3.17 (95% CI: 0.24; 6.11) and 1.47 (95% CI: 0.05; 2.88), respectively (Table 2). Regarding the glycemic profile, there was a negative association between *Is* levels and all glycemic parameters in Model 1; after including BMI and CRP (Model 2), the association was maintained for tertile 3 of fasting glycemia (β coef.: –1.81 (95% CI: –2.63; –0.98)) and HbA1c (β coef.: –0.06 (95% CI: –0.09; –0.03)) (Table 2).

**Table 2:** Association between serum iron and ferritin tertiles and cardiometabolic parameters in children aged 9–10 years

**Serum iron (μg/mL)**	**Model 1**^**a**^			**Model 2**^**b**^		
	**Tertile 1**	**Tertile 2**	**Tertile 3**	**Tertile 1**	**Tertile 2**	**Tertile 3**
	**β Coef. (95% CI)**					
*Lipid profile (n = 1954)*						
Total cholesterol (mg/dL)	(ref)	5.62 (2.81; 8.44)^**^	4.43 (1.57; 8.44)^*^	(ref)	4.68 (1.82; 7.53)^*^	3.17 (0.24; 6.11)^*^
HDL cholesterol (mg/dL)	(ref)	**1.07 (–0.37; 2.50)**	3.68 (2.22; 5.14)^**^	(ref)	–0.15 (–1.52; 1.23)	1.47 (0.05; 2.88)^*^
LDL cholesterol (mg/dL)	(ref)	4.59 (2.18; 7.01)^**^	1.49 (–0.96; 3.94)	(ref)	4.53 (2.08; 6.98)^**^	1.70 (–0.82; 4.21)
Triglycerides (mg/dL)	(ref)	–0.18 (–3.56; 3.20)	–3.77 (–7.20; –0.34)^*^	(ref)	1.47 (–1.79; 4.74)	0.02 (–3.38; 3.33)
*Glycemic profile*						
Fasting glucose (mg/dL) *(n = 1951)*	(ref)	–1.33 (–2.13; –0.53)^*^	–1.73 (–2.54; –0.92)^**^	(ref)	–1.49 (–2.30; –0.69)^**^	–1.81 (–2.63; –0.98)^**^
Glycated hemoglobin (%) *(n = 1946)*	(ref)	**–0.02 (–0.05; 0.01)**	–0.07 (–0.09; –0.04)^**^	(ref)	–0.02 (–0.05; 0.01)	–0.06 (–0.09; –0.03)^**^
Insulin (μU/mL) *(n = 1913)*	(ref)	–0.87 (–1.70; –0.04)^*^	–2.00 (–2.84; –1.16)^**^	(ref)	–0.31 (–1.08; 0.45)	–0.77 (–1.56; 0.02)
HOMA–IRc (µU/dL)^c^ *(n = 1910)*	(ref)	–0.22 (– 0.42; –0.01)^*^	–0.43 (–0.63; –0.22)^**^	(ref)	–0.10 (–0.29; 0.09)	–0.17 (–0.36; 0.03)
*Blood pressure (n = 1952)*						
Systolic pressure (mmHg)	(ref)	–1.38 (– 2.58; –0.19)^*^	–1.96 (–3.17; –0.74)^*^	(ref)	–0.68 (–1.79; 0.42)	–0.28 (–1.41; 0.86)
Diastolic pressure (mmHg)	(ref)	–0.98 (–1.95; 0.02)	–0.51 (–1.50; 0.47)	ref)	–0.61 (–1.54; 0.32)	0.45 (–0.50; 1.41)
**Serum ferritin (ng/mL)**	**Model 1**			**Model 2**		
	**Tertile 1**	**Tertile 2**	**Tertile 3**	**Tertile 1**	**Tertile 2**	**Tertile 3**
*Lipid profile (n = 1954)*						
Total cholesterol (mg/dL)	(ref)	1.62 (–1.21; 4.46)	1.66 (–1.17; 4.49)	(ref)	1.92 (–0.91; 4.74)	3.05 (0.18; 5.93)^*^
HDL cholesterol (mg/dL)	(ref)	–0.37 (–1.82; 1.07)	–3.99 (–5.42; –2.55)^**^	(ref)	–0.02 (–1.37; 1.34)	–2.23 (–3.61; –0.85)^*^
LDL cholesterol (mg/dL)	(ref)	2.03 (–0.39; 4.45)	5.08 (2.67; 7.50^**^	(ref)	2.06 (–0.336; 4.47)	5.30 (2.84; 7.76)^**^
Triglycerides (mg/dL)	(ref)	–0.02 (–3.41; 3.37)	2.71 (–0.67; 6.09)	(ref)	–0.71 (–3.63; 2.51)	–0.23 (–3.51; 3.05)
*Glycemic profile*						
Fasting glucose (mg/dL) *(n = 1951)*	(ref)	–0.65 (–1.45; 0.16)	–0.35 (–1.15; –0.46)	(ref)	–0.66 (–1.46; 0.14)	–0.37 (–1.19; 0.44)
Glycated hemoglobin (%) *(n = 1946)*	(ref)	–0.02 (–0.05; 0.01)	–0.04 (–0.07; –0.01)^*^	(ref)	–0.02 (–0.05; 0.01)	–0.05 (–0.08; –0.02)^*^
Insulin (μU/mL) *(n = 1913)*	(ref)	–0.23 (–1.07; 0.60)	1.01 (0.18; 1.85)^*^	(ref)	–0.47 (–1.22; 0.29)	–0.01 (–0.76; 0.78)
HOMA–IRc (µU/dL) *(n= 1910)*	(ref)	–0.07 (–0.27; 0.13)	0.23 (0.03; 0.43)^*^	(ref)	–0.12 (–0.31; 0.07)	0.02 (–0.18; 0.21)
*Blood pressure (n = 1952)*						
Systolic pressure (mmHg)	(ref)	0.26 (–0.94: 1.47)	1.51 (0.31; 2.71)^*^	(ref)	–0.07 (–1.16; 1.02)	0.16 (–0.95; 1.27)
Diastolic pressure (mmHg)	(ref)	0.18 (–0.80; 1.15)	1.35 (0.39; 2.32)^*^	(ref)	0.01 (–0.93; 0.93)	0.63 (–0.31; 1.58)

^*^p value < 0.05; ^**^p value < 0.001

^a^Model 1: β coefficient estimated by linear regression and adjusted for sex, age, family purchasing power, diet quality index (Med-DQI), dietary iron intake and physical activity (PAQ-C)

^b^Model 2: Model 1+ body mass index and C-reactive protein

^c^Homeostatic Model Assessment-Insulin Resistance

**Table 3:** Association between serum transferrin and transferrin saturation tertiles and cardiometabolic parameters in children aged 9 –10 years

	**Model 1**^**a**^			**Model 2**^**b**^		
**Transferrin (mg/dL)**	**Tertile 1**	**Tertile 2**	**Tertile 3**	**Tertile 1**	**Tertile 2**	**Tertile 3**
	**β Coef. (95% CI)**					
*Lipid profile *(*n *= 1954)						
Total cholesterol (mg/dL)	(ref)	5.91 (3.09; 8.73)^**^	8.81 (5.99; 11.63)^**^	(ref)	5.77 (2.95; 8.59)^**^	9.12 (6.26; 11.97)^**^
HDL cholesterol (mg/dL)	(ref)	1.48 (0.02; 2.94)	1.35 (–0.11; 2.81)	(ref)	1.85 (0.49; 3.22)^*^	2.95 (1.57; 4.33)^**^
LDL cholesterol (mg/dL)	(ref)	3.29 (0.86; 5.73)^*^	4.66 (2.23; 7.10)^**^	(ref)	2.99 (0.55; 5.43)^*^	4.06 (1.59; 6.53)^*^
Triglycerides (mg/dL)	(ref)	5.59 (2.24; 8.95)^*^	13.81 (10.46; 17.16)^**^	(ref)	4.53 (1.31; 7.75)^*^	10.34 (7.09; 13.60)^**^
*Glycemic profile*						
Fasting glucose (mg/dL) *(n = *1950*)*	(ref)	0.94 (0.13; 1.74)^*^	1.60 (0.80; 2.40)^**^	(ref)	0.79 (–0.02; 1.59)	1.31 (0.50; 2.13)^*^
Glycated hemoglobin (%) *(n = 1946)*	(ref)	0.03 (–0.00; 0.06)	0. 04 (0.01; 0.07)^*^	(ref)	0.03 (–0.00; 0.06)	0.04 (0.01; 0.06)^*^
Insulin (μU/mL) *(n = *1913*)*	(ref)	0.83 (–0.01; 1.67)	1.88 (1.04; 2.71)^**^	(ref)	0.47 (–0.29; 1.24)	0.69 (–0.08; 1.46)
HOMA–IR (µU/dL)^c^ *(n = *1910*)*	(ref)	0.18 (–0.02; 0.38)	0.40 (0.20; 0.60)^**^	(ref)	0.10 (–0.09; 0.30)	0.15 (–0.04; 0.34)
*Blood pressure *(*n* = *1952*)						
Systolic pressure (mmHg)	(ref)	0.44 (–0.77; 1.64)	2.69 (1.49; 3.89)^**^	(ref)	–0.17 (–1.27; 0.93)	0.85 (–0.26; 1.96)
Diastolic pressure (mmHg)	(ref)	0.61 (–0.36; 1.59)	1.48 (0.51; 2.46)^*^	(ref)	0.22 (–0.72; 1.15)	0.35 (–0.59; 1.29)
**Transferrin saturation (%)**	**Model 1**			**Model 2**		
	**Tertile 1**	**Tertile 2**	**Tertile 3**	**Tertile 1**	**Tertile 2**	**Tertile 3**
*Lipid profile *(*n *= 1954)						
Total cholesterol (mg/dL)	(ref)	2.56 (–0.29; 5.41)	2.20 (–0.65; 5.06)	(ref)	1.43 (–1.47; 4.43)	0.77 (–2.17; 3.71)
HDL cholesterol (mg/dL)	(ref)	0.95 (–0.51; 2.40)	3.07 (1.61; 4.52)^**^	(ref)	–0.42 (–1.81; 0.97)	0.55 (–0.86; 1.96)
LDL cholesterol (mg/dL)	(ref)	2.45 (–0.01; 4.90)	0.55 (–1.90; 2.99)	(ref)	2.37 (–0.12; 4.85)	0.79 (–1.73; 3.31)
Triglycerides (mg/dL)	(ref)	–4.49 (–7.90; –1.08)^*^	–7.07 (–10.47; –3.66)^**^	(ref)	–2.72 (–6.02; 0.58)	–2.89 (–6.23; 0.46)
*Glycemic profile*						
Fasting glucose (mg/dL) *(n = *1951*)*	(ref)	–1.35 (–2.15; –0.54)^*^	–2.38 (–3.18; –1.58)^**^	(ref)	–1.51 (–2.32; –0.70)^**^	–2.44 (–3.26; –1.61)^**^
Glycated hemoglobin (%) *(n = *1946*)*	(ref)	–0.03 (–0.06; 0.00)	–0.08 (–0.11; –0.05)^**^	(ref)	–0.02 (–0.05; –0.00)	–0.07 (–0.10; –0.04)^**^
Insulin (μU/mL) *(n = *1913*)*	(ref)	–1.38 (–2.21; –0.54)^*^	–2.33 (–3.17; –1.50)^**^	(ref)	–0.76 (–1.53; 0.02)	–0.90 (–1.68; –0.11)^*^
HOMA–IR (µU/dL) *(n = *1910*)*	(ref)	–0.33 (–0.53; –0.13)^*^	–0.52 (–0.72; –0.32)^**^	(ref)	–0.19 (–0.38; –0.00)^*^	–0.21 (–0.41; –0.02)^*^
*Blood pressure *(*n *= 1952)						
Systolic pressure (mmHg)	(ref)	–1.05 (–2.26; 0.16)	–2.47 (–3.68; –1.26)^**^	(ref)	–0.21 (–1.33; 0.90)	–0.48 (–1.61; 0.65)
Diastolic pressure (mmHg)	(ref)	–0.78 (–1.76; 0.20)	–0.87 (–1.85; 0.11)	(ref)	–0.32 (–1.27; 0.62)	0.29 (–0.67; 1.25)

^*^p value < 0.05; ^**^p value < 0.001

^a^Model 1: β coefficient estimated by linear regression and adjusted for sex, age, family purchasing power, diet quality index (Med-DQI), dietary iron intake and physical activity (PAQ-C)

^b^Model 2: Model 1+ body mass index and C-reactive protein

^c^Homeostatic Model Assessment-Insulin Resistance

Children with high levels of *Fs* (tertile 3) had, in the model of maximum adjustment, higher levels of TC and LDL-Chol (β coef.: 3.05 (95% CI: 0.18, 5.93) and 5.30 (95% CI: 2.84, 7.76), respectively) and lower levels of HDL-Chol (β coef.: –2.23 (95% CI: –3.61; –0.85)). There was a negative correlation between *Fs* and the glycemic parameters; when adjusted for BMI and CRP, the correlation was maintained only for HbA1c (β coef.: –0.05 (95% CI: –0.08; –0.02)) (Table 2).

Regarding *Tf* (Table 3), there was a positive association between high levels of *Tf* (tertile 3) and the concentration of CT [β coef.: 9.12 (95% CI: 6.26; 11.97)], HDL-Chol [β coef.: 2.95 (95% CI: 1.57; 4.33)], LDL-Chol [β coef.: 4.06 (95% CI: 1.59; 6.53)], and TG [β coef.: 10.34 (95% CI: 7.09; 13.60)]. Additionally, high *Tf* tertiles were associated with a higher fasting glucose concentration [β coef.: 1.31 (95% CI: 0.50; 2.13)] and HbA1c [β coef.: 0.04 (95% CI: 0.01; 0.06)].

Finally, there was a negative relationship between high levels of *STf* and all glycemic profile parameters (Table 3), with lower concentrations of glucose (p < 0.001), HbA1c (p < 0.001), and insulin (p < 0.05) and lower HOMA-IR values (p < 0.05).

The associations of the cardiometabolic parameters and the *Is*, *Fs*, *Tf* and *STf* tertiles, with logarithmic transformation of the CMRFs (Tables S1 and S2 of the supplementary material), were similar.

Table S3 (supplementary material) shows the prevalence of cardiometabolic risk factors in children aged 9–10 years.

Tables 4 and 5 show associations, in terms of adjusted OR, between the tertiles of the iron parameters and the prevalence of CMRFs. For *Is*, in Model 2, for the high tertile, the OR of having high LDL-Chol was 1.82 (95% CI: 1.03; 3.22), and the OR for low HDL-Chol values was 0.34 (95% CI: 0.17; 0.67). The risks (OR) of prediabetes, high insulin and high HOMA-IR were lower for the upper *Is* tertiles than for the lowest tertile (Table 4).

**Table 4:** Association between serum iron and serum ferritin tertiles and cardiometabolic risk factors in children aged 9–10 years

	**Model 1**^**a**^			**Model 2**^**b**^		
**Serum Iron (μg/mL)**	**Tertile 1**	**Tertile 2**	**Tertile 3**	**Tertile 1**	**Tertile 2**	**Tertile 3**
	**OR (95% CI)**					
*Dyslipidemia* (*n* = 1954)	-					
High total cholesterol^c^	1 (ref)	1.46 (0.98; 2.19)	1.43 (0.95; 2.15)	1 (ref)	1.37 (0.91; 2.06)	1.31 (0.86; 2.00)
Low HDL cholesterol^d^	1 (ref)	0.32 (0.18; 0.56)^**^	0.23 (0.12; 0.43)^**^	1 (ref)	0.38 (0.21; 0.69)^*^	0.34 (0.17; 0.67)^*^
High LDL cholesterol^e^	1 (ref)	1.76 (1.01; 3.06)^*^	1.86 (1.07; 3.22)^*^	1 (ref)	1.75 (0.99; 3.09)^*^	1.82 (1.03; 3.22)^*^
High triglycerides^f^	1 (ref)	1.02 (0.73; 1.42)	0.62 (0.43; 0.91)^*^	1 (ref)	1.06 (0.74; 1.52)	0.79 (0.53; 1.18)
*Dysglycemia*						
Prediabetes (*n*= 1954^g^	1 (ref)	0.81 (0.55; 1.18)	0.59 (0.39; 0.89)^*^	1 (ref)	0.81 (0.55; 1.19)	0.63 (0.41; 0.97)^*^
High insulin (*n *= 1913)^h^	1 (ref)	0.77 (0.52; 1.12)	0.33 (0.20; 0.53)^**^	1 (ref)	0.89 (0.58; 1.36)	0.53 (0.31; 0.90)^*^
High HOMA–IR (*n *= 1910)^i^	1 (ref)	0.67 (0.46; 0.96)^*^	0.24 (0.15; 0.40)^**^	1 (ref)	0.75 (0.49; 1.13)	0.37 (0.22; 0.64)^**^
*Blood pressure *(*n* = 1952)						
High blood pressure^j^	1 (ref)	0.90 (0.64; 1.26)	0.76 (0.53; 1.08)	1 (ref)	0.99 (0.68; 1.42)	1.03 (0.70; 1.52)
**Serum ferritin (ng/mL)**	**Model 1**			**Model 2**		
	**Tertile 1**	**Tertile 2**	**Tertile 3**	**Tertile 1**	**Tertile 2**	**Tertile 3**
*Dyslipidemia* (*n* = 1954)	**-**					
High total cholesterol	1 (ref)	0.93 (0.62; 1.38)	1.17 (0.80; 1.71)	1 (ref)	0.94 (0.63; 1.40)	1.29 (0.87; 1.90)
Low HDL cholesterol	1 (ref)	0.78 (0.38; 1.57)	2.76 (1.59; 4.79)^**^	1 (ref)	0.72 (0.35; 1.48)	2.07 (1.16; 3.68)^*^
High LDL cholesterol	1 (ref)	1.01 (0.59; 1.74)	1.52 (0.92; 2.52)	1 (ref)	1.02 (0.59; 1.76)	1.61 (0.97; 2.69)
High triglycerides	1 (ref)	1.08 (0.85; 1.54)	1.21 (0.85; 1.71)	1 (ref)	1.03 (0.71; 1.50)	0.98 (0.67; 1.42)
*Dysglycemia*	-					
Prediabetes (*n* = 1954)	1 (ref)	1.54 (1.04; 2.29)^*^	1.13 (0.74; 1.72)	1 (ref)	1.51 (1.01; 2.25)^*^	1.01 (0.66; 1.55)
High insulin (*n *= 1913)	1 (ref)	1.06 (0.69; 1.63)	1.33 (0.89; 2.00)	1 (ref)	0.96 (0.60; 1.54)	0.80 (0.50; 1.28)
High HOMA–IR (*n *= 1910)	1 (ref)	1.02 (0.67; 1.56)	1.41 (0.95; 2.09)	1 (ref)	0.92 (0.58; 1.46)	0.87 (0.55; 1.37)
*Blood pressure (n = 1952)*						
High blood pressure	1 (ref)	1.24 (0.85; 1.79)	1.69 (1.19; 2.41)^*^	1 (ref)	1.20 (0.81; 1.77)	1.46 (1.01; 2.13)^*^

^*^p value < 0.05; ^**^p value < 0.001

^a^Model 1: Odd ratios estimated by logistic regression and adjusted for sex, age, family purchasing power, diet quality index (Med-DQI), dietary iron intake and physical activity (PAQ-C)

^b^Model 2: Model 1+ body mass index and C–reactive protein

^c^High total cholesterol was defined as TC ≥ 200 mg/dL

^d^Low HDL cholesterol was defined as HDL-Chol < 40 mg/dL

^e^High LDL cholesterol was defined as LDL ≥ 130 mg/dL

^f^High triglycerides was defined as TG ≥ 130 mg/dL

^g^Prediabetes was defined as glucose > 100 mg/dL or glycated hemoglobin (HbA1c) ≥ 5.7%

^h^High insulin was defined as insulin ≥ 15 µU/dL

^I^High HOMA–IR (Homeostatic Model Assessment–Insulin Resistance) was defied as HOMA-IR ≥ 3.16 µU/dL

^j^High blood pressure was defined as systolic or diastolic blood pressure above the 9^0t^h percentile

**Table 5:** Association between transferrin and transferrin saturation tertiles and cardiometabolic risk factors in children aged 9–10 years

	**Model 1**^**a**^			**Model 2**^**b**^		
**Transferrin (mg/dL)**	**Tertile 1**	**Tertile 2**	**Tertile 3**	**Tertile 1**	**Tertile 2**	**Tertile 3**
	**OR (95% CI)**					
* Dyslipidemia* (*n* = 1954)						
High total cholesterol^c^	1 (ref)	1.40 (0.93; 2.09)	1.53 (1.03; 2.28)^*^	1 (ref)	1.38 (0.92; 2.06)	1.55 (1.04; 2.32)^*^
Low HDL cholesterol^d^	1 (ref)	0.71 (0.40; 1.24)	0.85 (0.50; 1.46)	1 (ref)	0.72 (0.40; 1.30)	0.62 (0.34; 1.11)
High LDL cholesterol^e^	1 (ref)	0.81 (0.48; 1.35)	0.99 (0.60; 1.63)	1 (ref)	0.81 (0.48; 1.35)	1.02 (0.61; 1.69)
High triglycerides^f^	1 (ref)	1.56 (1.03; 2.35)^*^	3.08 (2.12; 4.49)^**^	1 (ref)	1.46 (0.95; 2.24)	2.32 (1.56; 3.45)^**^
*Dysglycemia*						
Prediabetes (*n* = 1954)^g^	1 (ref)	1.13 (0.74; 1.71)	1.37 (0.92; 2.04)	1 (ref)	1.07 (0.70; 1.64)	1.21 (0.81; 1.82)
High insulin (*n *= 1913)^h^	1 (ref)	1.41 (0.87; 2.29)	2.64 (1.70; 4.09)^**^	1 (ref)	1.33 (0.79; 2.24)	1.68 (1.03; 2.75)^*^
High HOMA–IR (*n *= 1910)^i^	1 (ref)	1.67 (1.04; 2.68)^*^	2.85 (1.83; 4.42)^**^	**1 (ref)**	**1.66 (0.99; 2.78)**	1.90 (1.16; 3.12)^*^
*Blood pressure* (*n* = 1952)						
High blood pressure^j^	1 (ref)	0.83 (0.57; 1.21)	1.48 (1.05; 2.07)^*^	1 (ref)	0.73 (0.49; 1.08)	1.02 (0.71; 1.47)
**Transferrin saturation (%)**	**Model 1**			**Model 2**		
	**Tertile 1**	**Tertile 2**	**Tertile 3**	**Tertile 1**	**Tertile 2**	**Tertile 3**
*Dyslipidemia* (*n* = 1954)						
High total cholesterol	1 (ref)	1.14 (0.76; 1.70)	1.22 (0.82; 1.80)	1 (ref)	1.05 (0.70; 1.58)	1.10 (0.74; 1.66)
Low HDL cholesterol	1 (ref)	0.32 (0.18; 0.57)^**^	0.27 (0.14; 0.49)^**^	1 (ref)	0.41 (0.23; 0.73)^*^	0.44 (0.23; 0.84)^*^
High LDL cholesterol	1 (ref)	1.44 (0.83; 2.52)	1.75 (1.02; 3.00)^*^	1 (ref)	1.42 (0.81; 2.51)	1.69 (0.97; 2.96)
High triglycerides	1 (ref)	0.71 (0.51; 0.99)^*^	0.53 (0.36; 0.75)^**^	1 (ref)	0.74 (0.51; 1.06)	0.69 (0.47; 1.02)
*Dysglycemia*						
Prediabetes (*n *= 1950)	1 (ref)	0.79 (0.54; 1.15)	0.48 (0.32; 0.74)^*^	1 (ref)	0.80 (0.55; 1.18)	0.53 (0.34; 0.82)^*^
High insulin (*n *= 1913)	1 (ref)	0.71 (0.49; 1.03)	0.27 (0.17; 0.45)^**^	1 (ref)	0.91 (0.59; 1.40)	0.48 (0.28; 0.82)^*^
High HOMA–IR (*n *= 1910)	1 (ref)	0.60 (0.41; 0.87)^*^	0.23 (0.14; 0.37)^**^	1 (ref)	0.73 (0.48; 1.11)	0.37 (0.22; 0.64)^**^
*Blood pressure (n = 1952)*						
High blood pressure	1 (ref)	0.95 (0.68; 1.33)	0.66 (0.46; 0.95)^*^	1 (ref)	1.10 (0.77; 1.59)	0.97 (0.65; 1.43)

^*^p value < 0.05; ^**^p value < 0.001

^a^Model 1: Odd ratios estimated by logistic regression and adjusted for sex, age, family purchasing power, diet quality index (Med-DQI), dietary iron intake and physical activity (PAQ-C)

^b^Model 2: Model 1+ body mass index and C–reactive protein

^c^High total cholesterol was defined as TC ≥ 200 mg/dL

^d^Low HDL cholesterol was defined as HDL-Chol < 40 mg/dL

^e^High LDL cholesterol was defined as LDL ≥ 130 mg/dL

^f^High triglycerides was defined as TG ≥ 130 mg/dL

^g^Prediabetes was defined as glucose > 100 mg/dL or glycated hemoglobin (HbA1c) ≥ 5.7%;

^**h**^High insulin was defined as insulin ≥ 15 µU/dL

^I^High HOMA–IR (Homeostatic Model Assessment–Insulin Resistance) was defied as HOMA-IR ≥ 3.16 µU/dL

^j^High blood pressure was defined as systolic or diastolic blood pressure above the 90^th^ percentile

The association between *Fs* and having low HDL-Chol and high BP increased positively in tertile 3 compared to tertile 1, with ORs of 2.07 (95% CI: 1.16, 3.68) and 1.46 (OR 95% CI: 1.01, 2.13), respectively.

Compared with the participants in the lowest *Tf* tertile, the participants in the upper tertiles had a higher risk of high TC (OR: 1.55; 95% CI: 1.04; 2.32); high TG (OR: 2.32; 95% CI: 1.56; 3.45); high insulin (OR: 1.68; 95% CI: 1.03; 2.75) and high HOMA-IR values (OR: 1.90; 95% CI: 1.16; 3.12).

Participants in the upper *STf* tertiles had low HDL-Chol (OR: 0.44; 95% CI: 0.23; 0.84) compared with that of participants in the lower tertile. Additionally, the ORs for prediabetes, high insulin and high HOMA-IR were significantly lower for the participants in higher *STf,* tertiles, with ORs of 0.53 (95% CI: 0.34; 0.82), 0.48 (CI 95: 0.28; 0.82) and 0.37 (95% CI: 0.22; 0.64), respectively.

## Discussion

This study examined the relationship between different parameters of iron metabolism and CMRFs in a representative sample of children aged 9–10 years in Madrid (Spain). The direction and magnitude of the associations was variable depending on the biomarker analyzed. A worse lipid profile was observed with high *Fs* and *Tf* values, and *Is* was associated with an increase in both LDL-Chol and HDL-Chol. The glycemic profile was better in children who had high concentrations of *Is* and *STf* and was more unfavorable in children with high values of *Tf*. Finally, the only biomarker associated with an increased risk of hypertension was *Fs*.

Studies that have evaluated the relationship between iron parameters and CMRFs in the pediatric population are scarce [12–14, 28–31], and most have been carried out in Asian countries [12–14, 28, 29, 31]. The most widely used indicator of iron metabolism in these studies has been *Fs*, generally observing a worse lipid profile with higher concentrations of this biomarker. There are few disputes about the association between *Fs* and HDL-Chol levels, where there is a decrease in this cholesterol fraction when *Fs* levels are high [12, 13, 28, 30]; however, one study found a positive relationship, i.e., an increase in HDL-Chol [14]. The two studies that have examined the relationship between *Fs* and TC observed, as in this study, a positive association [12, 13]. There are more discrepancies in the association between *Fs* and TG; although a large number of studies found no association [12, 14, 30], two other studies observed contradictory associations, with one reporting a positive correlations [28] and the other reporting a negative correlation [13]. Few authors have incorporated other indicators of iron metabolism apart from *Fs*. Lee et al. studied *Is* and *ST* [12], and Zhang et al. studied *Is* [14], observing, as in our study, an increase in HDL-Chol with an increase in the levels of these biomarkers.

The evidence of associations between iron indicators and glycemic metabolism is also highly variable depending on the biomarkers used for evaluation. Three studies [12, 14, 30] report no association between *Fs* and fasting blood glucose, similar to the results of our study; however, in another work, higher concentrations of *Fs* were related to decreases in glucose levels [28]. Likewise, a majority of studies did not find an association between *Fs* and the HOMA-IR index [12, 13, 29]; however, one study reported a positive relationship [30]. Associations with other indicators of iron metabolism were similar between those observed in our study and those estimated by Lee et al., i.e., a decrease in blood glucose, insulin and HOMA-IR index with an increase in *Is* and *STf* [12], and a positive relationship between these glycemic indicators and *Tf* [29].

Regarding blood pressure, the increased risk of elevated blood pressure in children with high concentrations of *Fs* is consistent with the results reported by Yi et al. in a study of a male child population [28]; another study found no associations with *Fs* but rather with *Is* [14].

The mechanisms to explain the correlation between iron metabolism and lipid and glycemic metabolism are very complex and are related to the activation of oxidative stress factors, the response to inflammatory hypoxia and cell proliferation [32]. Excessive iron is sequestered by macrophages and deposited in muscles and adipose tissue, accelerating lipolysis, which affects the oxidation of lipoproteins, glucose and IR [33, 34]. This tissue damage also includes blood vessels, resulting in hypertension and vascular damage. In addition, high levels of iron generate high oxidative activity in the pancreas that can damage β cells, thus affecting insulin production [35]. Our results support these mechanisms, i.e., high levels of iron storage markers alter the lipid profile. In contrast, high levels of *Is, Tf and STf* activate the production of HDL-Chol, which can serve as a protective factor against oxidative stress in tissues and glucose metabolism [34]. The mechanisms underlying the negative association between *Is* and *STf* and glycemic metabolism and IR remain to be determined. High levels of ferritin and transferrin could antagonize the effect of insulin and contribute to IR [34]. Low levels of *Is* and *STf*, indicators of functional iron deficiency [36], can cause hepcidin dysregulation, resulting in iron being retained in reticulocytes, altering glucose metabolism and causing IR [37].

The levels of iron parameters fluctuate with age and can be altered through inflammatory processes, with certain chronic diseases and states of obesity causing chronic inflammation [2]. In this study, when introducing BMI and CRP differently from other covariates, the magnitude of the association was generally attenuated, potentially indicating a mediation effect; however, an independent effect remains.

### Strengths and limitations

To ensure the correct interpretation of the results of this study, some limitations and strengths should be considered. First, given the cross-sectional nature of the study design, the underlying causal sequence of the relationships between iron metabolism and CMRFs cannot be identified. Second, the data used to define high blood pressure were based on 1 measurement and not on 3 measurements taken on different days, as recommended by the European Society of Hypertension [24]. Third, although our models include the main covariates adjustment, some residual confounders cannot be ruled out.

Regarding strengths, various indicators of iron metabolism were available. In addition, each CMRF was analyzed independently to better understand its relationship with iron parameters. Second, this is one of the few studies conducted with a representative sample of the child population. Third, the main confounding variables were taken into account, including diet, with the quantification of iron intake, and physical activity. Finally, to control the independent effect of inflammatory mechanisms related to excess weight, BMI was included, as was CRP, in the regression models sequentially.

## Conclusions

The results of this study show that indicators of iron metabolism are associated with cardiometabolic alterations, with a direction and magnitude that vary depending on the biomarker analyzed. High concentrations of *Fs* and *Tf* are associated with a worse lipid profile, and *Is* is associated with an increase in both LDL-Chol and HDL-Chol. The glycemic profile is better in children who have high concentrations of *Is* and *STf* and is more unfavorable in children with high concentrations of *Tf*. The only biomarker positively associated with an increased risk of hypertension was *Fs*. Iron parameters in the pediatric population could be of great help to detect cardiometabolic alterations early.

### Supplementary Information

Below is the link to the electronic supplementary material.

Supplementary file1 (PDF 686 KB)

Supplementary file2 (PDF 589 KB)

Supplementary file3 (PDF 615 KB)

## Data Availability

According to private and confidential clauses stated in the informed consent, the dataset generated and analyzed during the current study is restricted and not publicly available for ethical reasons. Data will be available from the corresponding author (Dr. Honorato Ortiz-Marrón; E-mail: honorato.ortiz@salud.madrid.org) on reasonable request.

## Abbreviations

BMI: Body Mass Index
BP: Blood Pressure
CMRFs: Cardiometabolic risk factors
CI: Confidence Interval
ELOIN: Longitudinal Childhood Obesity Study
Fs: Serum ferritin
HbA1c: Glycated haemoglobin
HDL-Chol: High-Density Lipoprotein Cholesterol
HOMA-IR: Homeostatic Model Assessment—Insulin Resistance
Is: Serum iron
LDL-Chol: Low-Density Lipoprotein Cholesterol
MetS: Metabolic Syndrome
OR: Odds Ratio
SD: Standard Deviation
STf: Transferrin saturation
TC: Total Cholesterol
Tf: Transferrin
TG: Triglycerides
