# Supplementary material for: Association between biomarkers of iron status and cardiometabolic risk in Spanish children aged 9–10 years. The ELOIN study
Source: Eur J Pediatr. 2023 Oct 11;182(12):5649–59. doi: 10.1007/s00431-023-05244-1 (PMC10746575; doi:10.1007/s00431-023-05244-1)
Supplement: Supplementary file 1 — Supplementary file1 (PDF 686 KB) [file 431_2023_5244_MOESM1_ESM.pdf]

SUPPLEMENTARY INFORMATION

Table S1 Association between serum iron and serum ferritin tertiles and cardiometabolic parameters (with logarithmic transformation) in children aged 9–10 years

**Serum Iron (µg/mL)**	**Model 1**			**Model 2**		
	**Tertile 1**	**Tertile 2**	**Tertile 3**	**Tertile 1**	**Tertile 2**	**Tertile 3**
	**Exp(β)[b] (IC 95%)**					
Lipid profile (n = 1954)						
Total cholesterol (mg/dL)	1 (ref)	**1.04 (1.02; 1.05)†**	**1.03 (1.01; 1.05)†**	1 (ref)	**1.03 (1.01; 1.05)†**	**1.02 (1.00; 1.04)†**
HDL cholesterol (mg/dL)	1 (ref)	**1.03 (1.00; 1.05)†**	**1.07 (1.04; 1.10)††**	1 (ref)	1.00 (0.98; 1.03)	**1.03 (1.00; 1.05)†**
LDL cholesterol (mg/dL)	1 (ref)	**1.06 (1.03; 1.08)††**	1.02 (0.99; 1.05)	1 (ref)	**1.05 (1.02; 1.08)††**	1.02 (0.99; 1.05)
Triglycerides (mg/dL)	1 (ref)	1.01 (0.97; 1.05)	0.97 (0.93; 1.01)	1 (ref)	1.03 (0.99; 1.07)	1.02 (0.97; 1.06)
Glycemic profile						
Fasting glucose (mg/dL) *(n = 1951)*	1 (ref)	**0.98 (0.98; 0.99)†**	**0.98 (0.97; 0.99)††**	1 (ref)	**0.98 (0.97; 0.99)††**	**0.98 (0.97; 0.99)††**
Glycated haemoglobin (%) *(n = 1946)*	1 (ref)	1.00 (0.99; 1.00)	**0.99 (0.98; 0.99)††**	1 (ref)	1.00 (0.99; 1.00)	**0.99 (0.98; 0.99)††**
Insulin (µU/mL) *(n = 1913)*	1 (ref)	**0.86 (0.81; 0.93)††**	**0.76 (0.70; 0.81)††**	1 (ref)	**0.90 (0.85; 0.96)†**	**0.85 (0.80; 0.91)††**
HOMA–IRc (µU/dL)[c] *(n = 1910)*	1 (ref)	**0.85 (0.78; 0.91)††**	**0.74 (0.69; 0.80)††**	1 (ref)	**0.89 (0.83; 0.95)††**	**0.83 (0.78; 0.89)††**
Blood pressure (n = 1952)						
Systolic pressure (mmHg)	1 (ref)	**0.99 (0.97; 1.00)†**	**0.98 (0.97; 0.99)†**	1 (ref)	0.99 (0.98; 1.00)	1.00 (0.99; 1.01)
Diastolic pressure (mmHg)	1 (ref)	0.98 (0.97; 1.00)	0.99 (0.98; 1.01)	1 (ref)	0.99 (0.97; 1.01)	1.01 (0.99; 1.02)

**Serum ferritin (ng/mL)**	**Model 1**			**Model 2**		
	**Tertile 1**	**Tertile 2**	**Tertile 3**	**Tertile 1**	**Tertile 2**	**Tertile 3**
Lipid profile (n = 1954)						
Total cholesterol (mg/dL)	1 (ref)	1.01 (0.99; 1.03)	1.01 (0.99; 1.03)	1 (ref)	1.01 (1.00; 1.03)	1.02 (1.00; 1.04)
HDL cholesterol (mg/dL)	1 (ref)	0.99 (0.97; 1.02)	**0.93 (0.91; 0.95)††**	1 (ref)	1.00 (0.98; 1.02)	**0.96 (0.94; 0.98)††**
LDL cholesterol (mg/dL)	1 (ref)	**1.02 (1.00; 1.05)†**	**1.05 (1.03; 1.08)††**	1 (ref)	1.02 (1.00; 1.05)	**1.06 (1.03; 1.09)††**
Triglycerides (mg/dL)	1 (ref)	1.00 (0.96; 1.05)	1.03 (0.99; 1.08)	1 (ref)	1.00 (0.96; 1.04)	1.00 (0.96; 1.04)
Glycemic profile						
Fasting glucose (mg/dL) *(n = 1951)*	1 (ref)	0.99 (0.98; 1.00)	1.00 (0.99; 1.01)	1 (ref)	0.99 (0.98; 1.00)	1.00 (0.98; 1.01)
Glycated haemoglobin (%) *(n = 1946)*	1 (ref)	1.00 (0.99; 1.00)	**0.99 (0.99; 1.00)†**	1 (ref)	1.00 (0.99; 1.00)	**0.99 (0.99; 1.00)††**
Insulin (µU/mL) *(n = 1913)*	1 (ref)	0.99 (0.92; 1.06)	1.07 (1.00; 1.15)	1 (ref)	0.96 (0.90; 1.02)	0.97 (0.91; 1.03)
HOMA–IRc (µU/dL) *(n = 1910)*	1 (ref)	0.97 (0.90; 1.05)	1.06 (0.98; 1.14)	1 (ref)	0.95 (0.89; 1.02)	0.96 (0.90; 1.03)
Blood pressure (n = 1952)						
Systolic pressure (mmHg)	1 (ref)	1.00 (0.99; 1.01)	1.02 (1.00; 1.03)	1 (ref)	1.00 (0.99; 1.01)	1.00 (0.99; 1.01)
Diastolic pressure (mmHg)	1 (ref)	1.00 (0.98; 1.02)	**1.02 (1.00; 1.04)†**	1 (ref)	1.00 (0.98; 1.01)	1.01 (0.99; 1.03)

Model 1: coefficient of the generalized linear multivariate model adjusted for sex, age, family purchasing power, diet quality index (Med-DQI), dietary iron intake and Physical Activity (PAQ-C)

Model 2: Model 1+ body mass index and C–reactive protein

[b] Exponentiated β coefficient: represents the relative change in geometric means with respect to Tertile 1. Expressed as a percentage and interpreted as the percentage change in the geometric mean.

[c] Homeostatic Model Assessment–Insulin Resistance

† *p* value <0.05; †† *p* value <0.001
