# Supplementary material for: Association between biomarkers of iron status and cardiometabolic risk in Spanish children aged 9–10 years. The ELOIN study
Source: Eur J Pediatr. 2023 Oct 11;182(12):5649–59. doi: 10.1007/s00431-023-05244-1 (PMC10746575; doi:10.1007/s00431-023-05244-1)
Supplement: Supplementary file 2 — Supplementary file2 (PDF 589 KB) [file 431_2023_5244_MOESM2_ESM.pdf]

SUPPLEMENTARY INFORMATION

Table S2 Association between transferrin and transferrin saturation tertiles and cardiometabolic parameters (with logarithmic transformation) in children aged 9–10 years

	Model 1[a]			Model 2[b]		
**Transferrin (mg/dL)**	**Tertile 1**	**Tertile 2**	**Tertile 3**	**Tertile 1**	**Tertile 2**	**Tertile 3**
	**Exp(β)[c] (IC 95%)**					
Lipid profile (n = 1954)						
Total cholesterol (mg/dL)	1 (ref)	**1.04 (1.02; 1.06)[††]**	1.06 (1.04; 1.06)[††]	1 (ref)	**1.04 (1.02; 1.06)[††]**	**1.06 (1.04; 1.08)[††]**
HDL cholesterol (mg/dL)	1 (ref)	1.02 (1.00; 1.05)	1.02 (1.00; 1.05)	1 (ref)	**1.03 (1.01; 1.05)[†]**	**1.05 (1.03; 1.07)[††]**
LDL cholesterol (mg/dL)	1 (ref)	**1.04 (1.01; 1.07)[†]**	1.06 (1.03; 1.09)[††]	1 (ref)	**1.04 (1.01; 1.07)[†]**	**1.05 (1.02; 0.08)[†]**
Triglycerides (mg/dL)	1 (ref)	**1.08 (1.03; 1.12)[†]**	1.20 (1.15; 1.25)[††]	1 (ref)	**1.06 (1.02; 1.11)[†]**	**1.15 (1.10; 1.20)[††]**
Glycemic profile						
Fasting glucose (mg/dL) *(n = 1951)*	1 (ref)	**1.01 (1.00; 1.02)[†]**	**1.02 (1.00; 1.02)[††]**	1 (ref)	**1.01 (1.00; 1.02)[†]**	**1.02 (1.01; 1.03)[†]**
Glycated haemoglobin (%) *(n = 1946)*	1 (ref)	1.01 (1.00; 1.01)	**1.01 (1.00; 1.01)[†]**	1 (ref)	1.01 (1.00; 1.01)	**1.01 (1.00; 1.01)[†]**
Insulin (µU/mL) *(n = 1913)*	1 (ref)	**1.14 (1.06; 1.22)[†]**	**1.29 (1.20; 1.39)[††]**	1 (ref)	**1.09 (1.02; 1.16)[†]**	**1.14 (1.07; 1.21)[††]**
HOMA–IRc (µU/dL)[d] *(n = 1910)*	1 (ref)	**1.15 (1.07; 1.25)[††]**	**1.32 (1.22; 1.43)[††]**	1 (ref)	**1.11 (1.03; 1.18)[†]**	**1.16 (1.08; 1.24)[††]**
Blood pressure (N = 1952)						
Systolic pressure (mmHg)	1 (ref)	1.00 (0.99; 1.02)	**1.03 (1.01; 1.04)[†]**	1 (ref)	1.00 (0.99; 1.01)	1.01 (1.00; 1.02)
Diastolic pressure (mmHg)	1 (ref)	1.01 (0.99; 1.03)	1.03 (1.01; 1.04)	1 (ref)	1.00 (0.99; 1.02)	1.01 (0.99; 1.02)

	Modelo 1			Modelo 2		
**Transferrin saturation (%)**	**Tertile 1**	**Tertile 2**	**Tertile 3**	**Tertile 1**	**Tertile 2**	**Tertile 3**
Lipid profile (n = 1954)						
Total cholesterol (mg/dL)	1 (ref)	**1.02 (1.00; 1.03)[†]**	1.01 (1.00; 1.03)	1 (ref)	1.01 (0.99; 1.03)	1.01 (0.99; 1.02)
HDL cholesterol (mg/dL)	1 (ref)	**1.02 (1.00; 1.05)[†]**	**1.06 (1.03; 1.08)[††]**	1 (ref)	1.00 (0.98; 1.02)	1.01 (0.99; 1.04)
LDL cholesterol (mg/dL)	1 (ref)	**1.03 (1.00; 1.06)[†]**	1.00 (0.98; 1.03)	1 (ref)	**1.03 (1.00; 1.06)[†]**	1.01 (0.98; 1.04)
Triglycerides (mg/dL)	1 (ref)	**0.95 (0.91; 0.99)[†]**	**0.92 (0.88; 0.96)[††]**	1 (ref)	0.98 (0.94; 1.02)	0.98 (0.93; 1.02)
Glycemic profile						
Fasting glucose (mg/dL) *(n = 1951)*	1 (ref)	**0.98 (0.98; 0.99)[†]**	**0.97 (0.96; 0.98)[††]**	1 (ref)	**0.98 (0.97; 0.99)[††]**	**0.97 (0.96; 0.98)[††]**
Glycated haemoglobin (%) *(n = 1946)*	1 (ref)	1.00 (0.99; 1.00)	**0.99 (0.98; 0.99)[††]**	1 (ref)	1.00 (0.99; 1.00)	**0.99 (0.98; 0.99)[††]**
Insulin (µU/mL) *(n = 1913)*	1 (ref)	**0.85 (0.79; 0.91)[††]**	**0.73 (0.68; 0.79)[††]**	1 (ref)	**0.89 (0.84; 0.95)[†]**	**0.84 (0.79; 0.90)[††]**
HOMA–IRc (µU/dL) *(n = 1910)*	1 (ref)	**0.83 (0.77; 0.90)[††]**	**0.71 (0.66; 0.77)[††]**	1 (ref)	**0.88 (0.82; 0.94)[††]**	**0.82 (0.76; 0.87)[††]**
Blood pressure (N = 1952)						
Systolic pressure (mmHg)	1 (ref)	0.99 (0.98; 1.00)	**0.98 (0.96; 0.99)[††]**	1 (ref)	1.00 (0.99; 1.01)	1.00 (0.98; 1.01)
Diastolic pressure (mmHg)	1 (ref)	0.99 (0.97; 1.00)	0.99 (0.97; 1.00)	1 (ref)	0.99 (0.98; 1.01)	1.00 (0.99; 1.02)

[a]Model 1: coefficient of the generalized linear multivariate model adjusted for sex, age, family purchasing power, diet quality index (Med-DQI), dietary iron intake and Physical Activity (PAQ-C)

[b]Model 2: Model 1+ body mass index and C–reactive protein

[c]Exponentiated β coefficient: represents the relative change in geometric means with respect to Tertile 1. Expressed as a percentage and interpreted as the percentage change in the geometric mean.

[d]Homeostatic Model Assessment–Insulin Resistance

[†] *p* value <0.05; [††] *p* value <0.001
