# Supplementary material for: Association between biomarkers of iron status and cardiometabolic risk in Spanish children aged 9–10 years. The ELOIN study
Source: Eur J Pediatr. 2023 Oct 11;182(12):5649–59. doi: 10.1007/s00431-023-05244-1 (PMC10746575; doi:10.1007/s00431-023-05244-1)
Supplement: Supplementary file 3 — Supplementary file3 (PDF 615 KB) [file 431_2023_5244_MOESM3_ESM.pdf]

SUPPLEMENTARY INFORMATION

Table S3. Prevalence of cardiometabolic risk factors in children aged 9–10 years

Dyslipidemia **(*n* = 1954)**	**Prevalence (%)**	**(95% CI)**
High total cholesterol [a]	8.80	(7.63–10.14)
Low HDL cholesterol [b]	4.14	(3.34–5.12)
High LDL cholesterol [c]	4.91	(4.03–5.96)
High triglycerides [d]	11.16	(9.83–12.63)
Dysglycaemia		
Prediabetes (*n* = 1954) [e]	8.29	(7.15–9.60)
High insulin (*n* = 1913) [f]	8.11	(6.66–9.42)
High HOMA–IR (*n* = 1910) [g]	8.49	(7.32–9.82)
Blood pressure **(n=1952)**		
High blood pressure [h]	11.22	(9.90–12.70)

[a] High total cholesterol was defined as a TC level ≥ 200 mg/dL
[b] Low HDL cholesterol was defined was defined as an HDL–Chol level < 40 mg/dL
[c] High LDL cholesterol was defined as an LDL level ≥ 130 mg/dL
[d] High triglycerides was defined as a TG level ≥ 130 mg/dL
[e] Prediabetes was defined as a glucose level > 100 mg/dL or glycated haemoglobin (HbA1c) level ≥ 5.7%
[f] High insulin was defined as an insulin level ≥ 15 µU/dL.
[g] High HOMA–IR (homeostatic model assessment–insulin resistance) was defined as a HOMA–IR level ≥ 3.16 µU/dL.
[h] High blood pressure was defined as a systolic or diastolic blood pressure above the 90th percentile.
